# Microstructural Evolution, Thermodynamics, and Kinetics of Mo-Tm_2_O_3_ Powder Mixtures during Ball Milling

**DOI:** 10.3390/ma9100834

**Published:** 2016-10-15

**Authors:** Yong Luo, Guang Ran, Nanjun Chen, Qiang Shen, Yaoli Zhang

**Affiliations:** College of Energy, Xiamen University, Xiamen 361102, China; lyandzcc920626@163.com (Y.L.); njchen@umich.edu (N.C.); shenqiang1989@126.com (Q.S.); zhangyl@xmu.edu.cn (Y.Z.)

**Keywords:** neutron absorber, Mo-based composites, ball milling, microstructure, thulium oxide

## Abstract

The microstructural evolution, thermodynamics, and kinetics of Mo (21 wt %) Tm_2_O_3_ powder mixtures during ball milling were investigated using X-ray diffraction and transmission electron microscopy. Ball milling induced Tm_2_O_3_ to be decomposed and then dissolved into Mo crystal. After 96 h of ball milling, Tm_2_O_3_ was dissolved completely and the supersaturated nanocrystalline solid solution of Mo (Tm, O) was obtained. The Mo lattice parameter increased with increasing ball-milling time, opposite for the Mo grain size. The size and lattice parameter of Mo grains was about 8 nm and 0.31564 nm after 96 h of ball milling, respectively. Ball milling induced the elements of Mo, Tm, and O to be distributed uniformly in the ball-milled particles. Based on the semi-experimental theory of Miedema, a thermodynamic model was developed to calculate the driving force of phase evolution. There was no chemical driving force to form a crystal solid solution of Tm atoms in Mo crystal or an amorphous phase because the Gibbs free energy for both processes was higher than zero. For Mo (21 wt %) Tm_2_O_3_, it was mechanical work, not the negative heat of mixing, which provided the driving force to form a supersaturated nanocrystalline Mo (Tm, O) solid solution.

## 1. Introduction

Control rods are very important in both operating and accident conditions because nuclear reactivity needs to be controlled to safely operate a nuclear reactor [1]. The advantages of using gray control rods to control nuclear reactivity are that they greatly reduce the daily interaction with reactor coolant, they distinctly simplify the chemical and fault-tolerant control system and its operation, significantly decrease the high expense for a short time change, and also make the nuclear reactor safer [2,3]. The key to gray control rods controlling and adjusting nuclear reactivity is in their internal neutron absorbers, which can be prepared from bulk with elemental boron, Dy_2_TiO_5_ materials, Ag-In-Cd alloys, tungsten and silver, and their alloys [4,5,6,7,8]. Initially, boron carbide and boron steels were used as neutron absorbers in Russian nuclear power water reactors such as the VVER-1000, VVER-440, and RBMK-1000 models [5]. However, large irradiation damage, such as from swelling and cracking, were induced by (n,a)-reactions, void formation and gas bubble growth. Ag-In-Cd alloys are usually strong neutron absorbers [7] and can be also used in gray control rods [8]. However, the nuclear reactivity value of gray control rod assemblies drops to approximately 80% of its initial value after running for five years, which no longer meets the control condition of the mechanical shim. Dy_2_TiO_5_ pellets are used as neutron absorbers in Russian thermal reactors, such as VVER-1000 RCCAs (Vodo-Vodyanoi Energetichesky Reaktor 1000 Rod Control Cluster Assembly) [5,6]. However, the neutron absorption ability decreases with increasing service time [9,10]. Other lanthanide elements, such as terbium, are needed as a reinforcing agent to balance the neutron absorption ability in order to maintain the stability of the nuclear reaction. 

According to nucleon calculation, nuclear property assessment, and burn-up analysis, thulium is an excellent candidate for use as a neutron absorber [8]. Natural thulium consists of one stable isotope, ^169^Tm. Both thulium decay products, Yb and Lu, and their offspring decay products, can absorb neutrons. The absorption cross-section of thulium is 105 barns, and the region of resonance absorption is 3.92–17.6 eV [9,10]. Theoretical calculation shows that the gray control rod assemblies of loaded neutron absorbers with thulium still have a nuclear reactivity value that is close to the initial value after a long burn-up time. Even if they are burned up for 20 years, the nuclear reactivity worth is changed only slightly. Therefore, it is important to develop and synthesize bulk material containing elemental thulium for use as neutron absorbers in gray control rods. According to in-service conditions in the nuclear reactor core, neutron absorbers should have high density, good thermophysical properties, sufficient physical efficiency, and excellent irradiation resistance. Meanwhile, the size factor, fabricability of neutron absorber pellets in a practical application, and linear density of thulium in pellets must also be considered. Therefore, Mo-based Tm_2_O_3_ composites are designed and considered as excellent neutron absorbers based on the excellent characteristics of Mo components, such as high melting temperature, high temperature strength, excellent thermal conductivity, and outstanding irradiation resistance. Meanwhile, the thermal neutron absorption cross-section of Mo (2.7 barns) is much smaller than that of Tm (105 barns), which has little effect on the behavior of nuclear characteristics of Tm during long burn-up times. In addition, there is no chemical reaction between Mo and Tm_2_O_3_ at temperatures below 1600 °C, which can ensure the stability of the phases of Mo-based Tm_2_O_3_ composites in service. However, almost no studies relating to their microstructure, thermophysical properties, physical efficiency, and irradiation behavior can be found at present.

High-energy ball milling is a non-equilibrium solid-state alloying technology for powder mixture preparations that can be used to synthesize novel materials impossible to obtain by conventional technology. It is well known that ball milling of powder mixtures can generate equilibrium and non-equilibrium structures, including supersaturated solid solutions, nanocrystalline powders, metastable compounds, and amorphous solids [11,12,13,14]. The grain size and the agglomeration degree of the mixture particles have a great influence on both the packing achievement in the green bodies and the final sintered density and grain size [15]. The nanocrystalline powders have excellent sintering ability, low sintering temperature, and a high tendency for increased density [16], which can improve the properties and performance of sintered bulks. 

Therefore, based on the defects of neutron absorbers, such as bulk with elemental boron, Dy_2_TiO_5_ materials, and Ag-In-Cd alloys, and the advantages of Mo and Tm_2_O_3_ components, the Mo-Tm_2_O_3_ composite will be an excellent candidate as a neutron absorber. In the present work, ball-milling technology was first used to prepare Mo-Tm_2_O_3_ powder mixtures. The microstructural evolution, thermodynamics, and kinetics of Mo-Tm_2_O_3_ powder mixtures during ball milling were investigated. The corresponding mechanism was also analyzed and discussed. The current experimental results of ball-milled powder mixtures can provide sufficient support for subsequent bulk preparation. 

## 2. Experiments

The powders of Mo (99.9% purity) and Tm_2_O_3_ (99.6% purity), with an average particle diameter of 60 μm and 5 μm, respectively, were used as raw materials. The raw powders of Tm_2_O_3_ and Mo were purchased from Beijing HWRK Chem Co., Ltd. of China (Beijing, China) and Jiangxi KETAI Advanced Material Co., Ltd. of China (Nanchang, China), respectively. Ball milling of Mo (21 wt %) Tm_2_O_3_ powder mixtures was carried out at room temperature on a SFM-1 high-energy planetary ball mill manufactured by Shenyang Kejing Auto-instrument Co., Ltd. (Shenyang, China). The mass fraction of Mo (21 wt %) Tm_2_O_3_ is equal to the molar fraction of Mo (6.2 at %) Tm_2_O_3_. Stainless steel balls of 5 mm in diameter were used as the milling media. The ball-to-powder mass ratio was 10:1 and the rotational speed was 500 rpm. No more than 1 wt % stearic acid was added in the powder mixtures as a process control agent to prevent excessive cold welding and aggregation amongst powder particles. During ball milling, a 5 min interval stop was used after running 55 min to prevent excess generated heat that had an obvious effect on the ball milling procedure. In order to avoid contamination, the powder mixtures were first milled at low speed for a few minutes, so a powder coating covered the stainless steel balls and vial wall. Meanwhile, the powder mixtures used for microstructure analysis were extracted from the loose powders in the steel can, not from the powders stuck on the stainless ball surface and the steel can wall surface. 

After ball milling over different time periods, small amounts of powder mixtures were taken from the container and characterized by X-ray diffraction (XRD) on a Rigaku D/max-3C X-ray diffractometer (Tokyo, Japan) with Cu Kα radiation (*λ* = 0.1540598 nm). The grain size was calculated using Suryanarayana and Grant Norton’s formula [17]:

(1) $$B_{r}\;\text{cos}\;\theta =\frac{K\lambda }{L}+\eta \;\text{sin}\;\theta$$

where *K* is a constant (with a value of 0.9); *λ* is the wavelength of the X-ray radiation; *L* and *η* are the grain size and internal strain, respectively; and *θ* is the Bragg angle. *B_r_* is the full width at half-maximum (FWHM) of the diffraction peak after instrumental correction and can be calculated from the following equation:

(2) *B* = *B_r_* + *B_s_*

where, *B* and *B_S_* are the FWHM of the broadened Bragg peaks and the standard sample’s Bragg peaks, respectively.

The microstructure of the ball-milled powders was analyzed by transmission electron microscopy (TEM) on a JEM-2100 instrument (JEOL, Tokyo, Japan). The ball-milled mixtures were first put in ethyl alcohol, and then adequately dispersed by ultrasonic vibration. A carbon-coated copper grid was used to collect the dispersed powders in the ethyl alcohol and then dried by an ultraviolet lamp. After that, the prepared samples were observed by the TEM instrument (JEOL, Tokyo, Japan).

## 3. Results and Discussion

### 3.1. Phase Evolution and Microstructure Analysis

**Figure 1:**
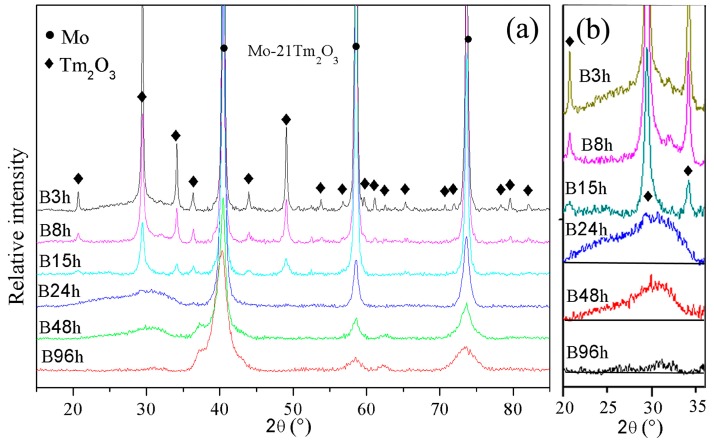
(**a**,**b**) X-ray patterns of Mo (21 wt %) Tm_2_O_3_ powder mixtures milled for different times at a 2θ of 15°–85° and 20°–36°, respectively.

The XRD patterns of Mo (21 wt %) Tm_2_O_3_ powder mixtures milled for different times are shown in Figure 1. The diffraction peaks of the Mo and Tm_2_O_3_ phases are broadened significantly and reduced in intensity with increasing milling time. Meanwhile, a small position shift of Mo peaks to a low diffraction angle is detected, which indicates the large-sized Tm atoms are dissolved into the Mo crystal structure. 

The diffraction peaks indicate that the Tm_2_O_3_ crystal structure disappears after 24 h of ball milling, which is due to the destruction of the Tm_2_O_3_ crystal structure by ball milling. Only a diffraction hill indicating Tm_2_O_3_ amorphization can be observed in the XRD spectrum as shown in Figure 1b. Meanwhile, the intensity of the diffraction hill decreases with increasing milling time. The diffraction hill disappears completely after 96 h of ball milling, which may be attributed to Tm and O atoms being dissolved into the Mo crystal structure. This result is similar with Zhang’s investigation that showed the intensity of Y_2_O_3_ diffraction peaks almost disappeared after 52 h of ball milling in a Co-based oxide dispersion strengthened alloy, and this was attributed to Y_2_O_3_ decomposition and dissolution into the Co crystal structure during ball milling [18]. The effect of ball milling is more evident in the Tm_2_O_3_ phase than the Mo phase. In addition, no diffraction peaks of new phases among Mo, Tm, and O elements are observed, which indicates that no intermetallic compounds are formed during ball milling. Ball milling does not induce a chemical reaction between Mo and Tm_2_O_3_, and the amount of intermetallic compounds formed between Mo and Tm_2_O_3_ is also negligible even if they had a reaction.

**Figure 2:**
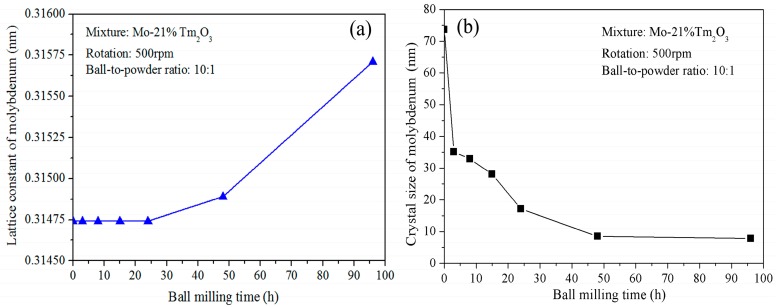
(**a**) Curve of the lattice parameter of Mo vs. ball-milling time; and (**b**) curve of the crystalline size of Mo vs. ball-milling time.

The broadening of X-ray diffraction peaks is associated with the refinement of the grain size and lattice distortion during ball milling. Figure 2a shows the variation of Mo lattice parameter with increasing ball-milling time. It is demonstrated that the lattice parameter increases from 0.31474 nm to 0.31564 nm as the ball-milling time increases from 0 to 96 h. In the initial stage, the Mo lattice parameter remains almost constant. After further ball milling, the Mo lattice parameter increases with increasing ball-milling time. The reason for the Mo lattice parameter increment can be attributed to the dissolution of Tm and O atoms. The supersaturated nanocrystalline solid solution of Mo (Tm, O) is formed after 96 h of ball milling in this system. This result differs from Raghavendra’s research, which showed that the diffraction peaks of the Fe and ZrO_2_ phases could be still observed after 100 h of ball milling in a Fe (15 wt %) ZrO_2_ system [19]. However, Toualbi reported the diffraction peak disappearance of the Y_2_O_3_ phase in a Fe (9 wt %)-Cr (10 wt %) Y_2_O_3_ system during ball milling was due to the dissolution of a very small number of yttria particles into the matrix to form a solid solution and to the amorphization of a large amount of Y_2_O_3_ particles [20]. 

Figure 2b shows the grain size of the ball-milled powders with increasing ball-milling time. It indicates that the refinement rate of crystallite size is roughly logarithmic with the ball-milling time. In fact, the grain size was calculated for the Mo phase, not for the Tm_2_O_3_ phase, because Tm_2_O_3_ peaks disappeared completely. It can be seen that ball milling results in a fast decrease of Mo grain size in the initial stage and a constant value in the later stage. The average grain size of the Mo phase is approximately 74 nm and 35 nm after ball milling of 0 and 3 h, respectively. The decrease rate is approximately 13 nm/h. After further ball milling from 3 to 24 h, the average grain size decreases from 35 to 17 nm. The decrease rate is approximately 0.85 nm/h. However, the average grain size only decreases from 17 to 8 nm after ball milling from 24 to 96 h. The decrease rate is only 0.125 nm/h, which indicates that further refinement of grain size occurs slowly after an extended milling time. In particular, Mo grain size remains almost constant at 8 nm from 48 to 96 h of ball milling. The lattice distortion calculated from the X-ray broadening exhibits an increasing tendency. The total lattice distortion is approximately 1.5% after 96 h of ball milling.

**Figure 3:**
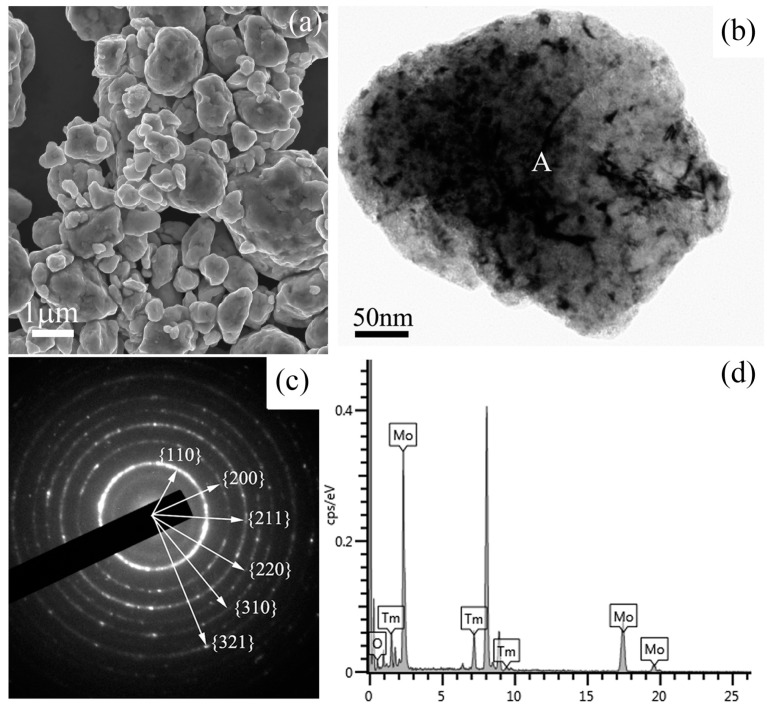
Microstructure analysis results of Mo (21 wt %) Tm_2_O_3_ powder mixtures milled for 48 h; (**a**) SEM image showing the morphology of the ball-milled powders; (**b**) bright field TEM image; (**c**) selected area electron diffraction pattern from region ‘A’ in (**a**) showing the Mo phase; and (**d**) EDS analysis results from region ‘A’ in (**a**) showing the diffraction peaks of Tm, Mo, O, and Cu elements. Cu peaks comes from the copper grid.

Figure 3 shows a SEM image, a bright field TEM image, the corresponding selected area electron diffraction (SAED) pattern, and the energy dispersive spectrometer (EDS) results of Mo (21 wt %) Tm_2_O_3_ powder mixtures milled for 48 h. The SEM image in Figure 3a shows the morphology of the ball-milled powder mixtures, which indicates that many particles with small size aggregate to form a large-size particle. The size of the particle is over one micrometer. A spherical particle with a size of approximately 500 nm can be observed in the TEM image. Some defects, such as dislocations, voids, and crystal boundaries can also be observed in this particle, which were induced by heavy deformation of powder mixtures during ball milling. These defects enhance the diffusivity of solute atoms into the Mo matrix. The SAED pattern taken from the region marked ‘A’ in Figure 3b is shown in Figure 3c. After analyzing and indexing the ring-shaped SAED pattern, it is indicated that this SAED pattern belongs to the Mo phase, not the Tm_2_O_3_ phase. Meanwhile, it is also demonstrated that the Mo component has already been nano-crystallized, which is also confirmed by the calculation results of Mo crystallites derived from Bragg diffraction peaks in Figure 1. Although the size of the particles is still at the micrometer scale, as shown in Figure 3a, the grain size is up to the nanometer scale. The EDS spectrum taken from the ball-milled particle is shown in Figure 3d, which includes Mo, O, and Tm element peaks. Similar EDS results are detected in other particles. Although the SAED results show that the particle belongs to a Mo crystal, Tm and O elements are also detected in this particle. Therefore, it indicates that Tm_2_O_3_ either uniformly distributes on the Mo particle surface in an amorphous form or dissolves into the Mo crystal structure in the form of Tm and O atoms. However, few diffraction halos related to the amorphous phase can be found in this SAED pattern, as show in Figure 3c, which indicates that Tm_2_O_3_ does not exist mainly in the amorphous form in this particle. 

**Figure 4:**
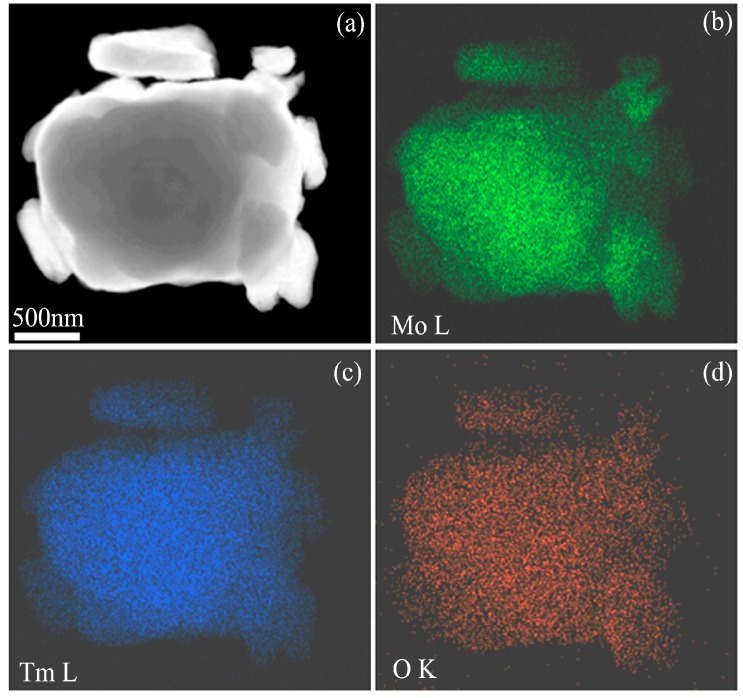
STEM analysis results of Mo (21 wt %) Tm_2_O_3_ powder mixtures milled for 96 h; (**a**) high angle annual dark field STEM image; (**b**–**d**) Mo L, Tm L, and O K element mappings, respectively.

Figure 4a is a high angle annual dark field STEM image of Mo (21 wt %) Tm_2_O_3_ powder mixtures milled for 96 h, which shows several small particles agglomerated to form a large-sized particle. Figure 4b–d are Mo L, Tm L, and O K element mappings of the particles in Figure 4a, respectively. It can be seen that Mo, Tm, and O atoms are uniformly distributed in the particles. Every location contains Mo, Tm, and O elements, which further demonstrates that Tm_2_O_3_ dissolves into the Mo crystal structure after 96 h of ball milling because the diffraction hill indicating Tm_2_O_3_ amorphization disappears completely after 96 h of ball milling, as shown in Figure 1b. 

In fact, the Tm_2_O_3_ component is a brittle powder, which is fragmented during ball milling. The particle size reduces continuously as a consequence of the energy provided by ball milling. However, the Mo component belongs to the ductile powder, which is repeatedly flattened, cold welded, fractured, and rewelded by the force of the impact. In the initial stage of ball milling, the microstructural evolution of this ductile-brittle Mo-Tm_2_O_3_ system is that the ductile Mo metal powder particles get flattened by the ball-powder-ball collisions, while the brittle Tm_2_O_3_ oxide get fragmented. These fragmented Tm_2_O_3_ particles tend to become occluded by the ductile Mo constituents and trapped in the ductile Mo particles. With further ball milling, Mo particles get work hardened, fractured, and refined. The newly created surface of the Mo component has a large number of dangling bonds that have high chemical activity, which makes the fined Mo particles aggregate together to form a large-sized particle, and also combined with dangling bonds of the fined Tm_2_O_3_ particles at an atomic level. Meanwhile, fragmented Tm_2_O_3_ particles will be continuously fined to smaller-sized particles with increasing ball-milling time and will be decreased to several nanometers. In particular, as the size of Tm_2_O_3_ is decreased to 1~2 nanometers, the ratio of atoms on the particle surface is very large. With increasing ball-milling time, Tm_2_O_3_ is transformed gradually from a crystal structure to an amorphous phase, as shown in Figure 1b. The amorphous atom arrangement is disordered over a long distance, but ordered over a very short distance, which could induce Tm and O atoms to attach to a Mo particle surface in a state of dissociation. Tm and O atoms can easily diffuse into the Mo crystal structure. Meanwhile, heavy deformation is introduced continually into Mo particles. This is manifested by the presence of a variety of crystal defects, such as dislocations, vacancies, stacking faults, and an increased number of crystal defect boundaries [21], which can enhance the diffusivity of Tm and O atoms into the Mo matrix. Moreover, the refined microstructural features decrease Tm and O atom diffusion distances. Additionally, the rise in temperature during ball milling further promotes diffusion. Consequently, Tm and O atoms dissolve into the Mo crystal structure. The longer the ball-milling time is, the greater the amount of dissolution of Tm atoms. After 96 h of ball milling, the supersaturated nanocrystalline solid solution of Mo (Tm, O) is formed. 

### 3.2. Thermodynamics Analysis 

As mentioned above, long time ball milling induces the Tm_2_O_3_ component to be decomposed and then dissolved into the Mo crystal structure. Therefore, thermodynamics will be used to analyze the dissolution behavior of Tm into the Mo crystal structure. 

Based on Miedema semi-experimental theory [22], the Gibbs free energy of a Mo-Tm solid solution is estimated as:

(3) $$\Delta G^{s}=\Delta H_{m}^{s}-T\Delta S^{s}$$

where $\Delta H_{m}^{s}$ and $\Delta S^{s}$ are mixing enthalpy and mixing entropy, respectively. With the assumption of an ideal solution, $\Delta S^{s}$ can be expressed as:

(4) $$\Delta S^{s}=-R(x_{i}\;\text{ln}\;x_{j}+x_{j}\;\text{ln}\;x_{i})$$

where $x_{i}$ and $x_{j}$ are the molar fractions of element A and B, $x_{i}$ + $x_{j}$ = 1; *R* is the gas constant; and *T* is the reaction temperature.

The Gibbs free energy, $\Delta G^{s}$, can be rewritten as:

(5) $$\Delta G^{s}=\Delta H_{m}^{s}+RT\left(x_{i}\;\text{ln}\;x_{i}+x_{j}\;\text{ln}\;x_{j}\right)$$

The enthalpy,$\Delta H_{m}^{s}$, of a solid solution formation can be written as [23]:

(6) $$\Delta H_{m}^{s}=\Delta H_{chem}+\Delta H_{Elast}+\Delta H_{Struct}$$

where $\Delta H_{Chem}$, $\Delta H_{Elast}$, and $\Delta H_{Struct}$ are the chemical, elastic, and structural contributions due to the mixing of the two different atoms, the atom size mismatch, and taking into account the difference in valence and crystal structure of the solute and solvent, respectively. Compared with the first two items, the structural contributions have only a minor effect [24], which will be ignored here. Thus $\Delta H_{m}^{s}$ will be rewritten as:

(7) $$\Delta H_{m}^{s}=\Delta H_{chem}+\Delta H_{Elast}$$

$\Delta H_{Chem}$ can be expressed by:

(8) $$\Delta H_{Chem}=x_{i}x_{j}\left(f_{j}^{i}\Delta H_{Sol}^{iinj}+f_{i}^{j}\Delta H_{Sol}^{jini}\right)$$

where $\Delta H_{Sol}^{iinj}$ is the solution enthalpy of *i* in *j* given by:

(9) $$\Delta H_{Sol}^{iinj}=\frac{2PV_{i}^{2/3}}{{\left(n_{ws}^{-1/3}\right)}_{i}+{\left(n_{ws}^{-1/3}\right)}_{j}}\left[-{\left(\Delta \phi^{*}\right)}^{2}+\frac{Q}{P}{\left(\Delta n_{ws}^{1/3}\right)}^{2}\right]$$

$f_{i}^{j}$ is the parameter of adjacent atom in solution solid and can be expressed as:

(10) $$f_{i}^{j}=C_{i}^{S}\left\lfloor 1+\delta {\left(C_{j}^{S}.C_{i}^{S}\right)}^{2}\right\rfloor$$

(11) CiS=xiVi2/3(xiVi2/3+xjVj2/3)

where $\Delta \phi^{*}$, V, and $n_{ws}$ are the work function, molar volume, and electron density of the constituents, respectively. *P* and *Q* are empirical constants having the same value for widely different metal combinations. δ is taken to be five for the short-range order and eight for the long-rang order, respectively.

$\Delta H_{Elast}$ can be expressed as:

(12) $$\Delta H_{Elast}=x_{i}x_{j}\left(x_{i}\Delta E_{e}^{iinj}+x_{j}\Delta E_{e}^{jini}\right)$$

where $\Delta E_{e}^{iinj}$ is the size mismatch contribution to the enthalpy of a solution of *i* and *j* per mol *i* and can be estimated as [25]:

(13) $$\Delta E_{e}^{iinj}=\frac{2K_{i}u_{j}{\left(V_{j}^{*}-V_{i}^{*}\right)}^{2}}{3K_{i}V_{j}^{*}+4u_{j}V_{i}^{*}}$$

where K, u, and $V^{*}$ are the bulk modulus, shear modulus, and atomic volume, respectively. $V^{*}$ can be expressed as:

(14) $${\left(V_{i}^{*}\right)}^{2/3}=V_{i}^{2/3}\left(1+cf_{j}^{i}\Delta \phi_{AB}\right)$$

where *c* is constant.

The Gibbs free energy of formation of an amorphous phase can be estimated by [26]:

(15) $$\Delta G^{Amorp.}=\Delta H^{Amorp.}-T\Delta S^{Amorp.}+x_{i}\Delta G_{i}^{Amorp.-Cryst.}+x_{j}\Delta G_{j}^{Amorp.-Cryst.}$$

where $\Delta H^{Amorp.}$ and $\Delta S^{Amorp.}$ are the enthalpy and entropy of mixing of the amorphous phase. $\Delta G_{i}^{Amorp.-Cryst.}$ is the difference in Gibbs free energy between the amorphous and crystalline phases of the pure element at room temperature and can be calculated according to the formula proposed by Thompson:

(16) ΔGi(j)Amorp.−Cryst.=2TΔHf(Tm−T)Tm(T+Tm)

where $\Delta H_{f}$ and *T_m_* are the enthalpy of fusion and melting temperature, respectively.

$\Delta H^{Amorp.}$ contains only the chemical contribution due to the amorphous structure, which can be calculated by:

(17) $$\Delta H^{Amorp.}=\Delta H_{Chem.}$$

(18) $$\Delta S^{Amorp.}=-R\left(x_{i}\;\text{ln}\;x_{i}+x_{j}\;\text{ln}\;x_{j}\right)$$

**Figure 5:**
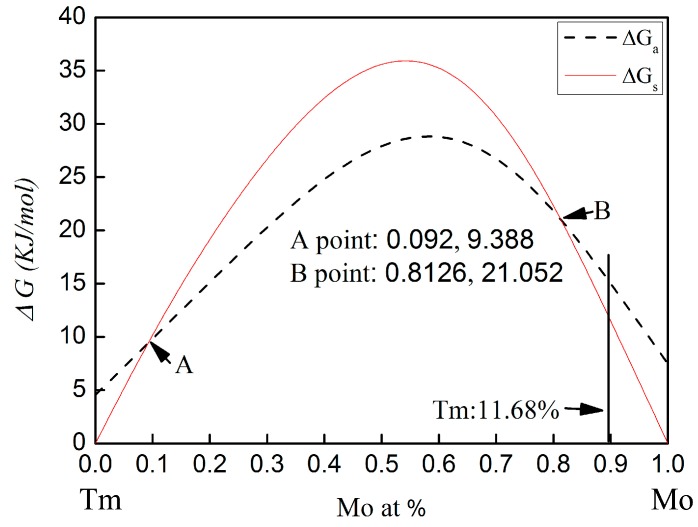
Gibbs free energy of the Mo-Tm system vs. molybdenum content. $\Delta G_{s}$ and $\Delta G_{a}$ are the Gibbs free energy of the crystalline solid solution and amorphization, respectively.

The calculated Gibbs free energy changes on forming an amorphous phase and crystalline solid solution in the Mo-Tm system is shown in Figure 5. The temperature for calculating the Gibbs free energy is taken to be 373 K [11]. The parameters used for calculation are listed in Table 1 [27,28]. The calculated Gibbs free energy of both the crystalline solid solution and the amorphous phase are higher than zero for all compositions. Therefore, there is no chemical driving force to form a crystalline solid solution or an amorphous phase. From Figure 5, it can be seen that, with molybdenum content in the range of 0~9.2 at % or 81.26~100 at %, the Gibbs free energy for the formation of an amorphous phase is higher than that of a crystalline solid solution. The Gibbs free energy curve of the crystalline solid solution intersects with that of the amorphous phase at the 9.2 at % and 81.26 at % Mo content.

In the present work, the molar percentage of Tm atoms is 11.68 at %. The Gibbs free energy for the formation of an amorphous phase and a crystalline solid solution is 16.36 KJ/mol and 13.5 KJ/mol, respectively. The Gibbs free energy of amorphization is higher than that of the crystalline solid solution formation. Therefore, the formation of the crystalline solid solution is energetically preferred over the amorphous phase. However, the Gibbs free energy of the formation of the crystalline solid solution and the amorphization are both higher than zero for this chemical composition. There is no chemical driving force to form a crystalline solid solution and an amorphous phase. Meanwhile, there is also no chemical driving force for the transformation from crystalline to amorphous in this chemical composition. To more deeply understand the mechanism for microstructural evolution of Mo-Tm_2_O_3_ powder mixtures, especially in regard to the dissolution of Tm atoms into the Mo crystal structure, the reaction kinetics during ball milling must be considered. 

In addition, although the calculated Gibbs free energy of both the crystalline solid solution and the amorphous phase are higher than zero for all compositions, as shown in Figure 5, these calculated values are limitations and cannot completely represent actual situation. However, ball milling induced the decomposition of the Tm_2_O_3_ component, followed by solvation into the Mo crystal structure, the investigation of the Gibbs free energy using the Mo-Tm system to take the place of Mo-Tm_2_O_3_ system is also reasonable and feasible. 

### 3.3. Dynamics Analysis 

The atomic radii of the Mo atom and Tm atom is 0.19 nm and 0.222 nm, respectively [29]. The difference in atomic radius is approximately 15%. At high pressure, the radii of the Mo atom and Tm atom will reach the same value. Under this condition, the Tm atom will easily dissolve into the Mo crystal structure. The required pressure is given by the following:

(19) *R*_Mo_·(1 − *ε*_Mo_) = *R*_Tm_·(1 − *ε*_Tm_)

where *ε*_Mo_ = q*/*E*_Mo_, *ε*_Tm_ = q*/*E*_Tm_. Here, *R* is the initial atom radius; q* is the pressure; *E* is the bulk elastic modulus; and *ε* is the amount of strain. Therefore, Equation (19) can be rewritten as follows:

(20) *R*_Mo_·(1 − q*/*E*_Mo_) = *R*_Tm_·(1 − q*/*E*_Tm_)

where *R*_Mo_ = 0.19 nm, *R*_Tm_ = 0.222 nm, *E*_Mo_ = 230 GPa, and *E*_Tm_ = 45 GPa. The q* value can be obtained according to Equation (20) and is q* = 7.79 GPa. Therefore, when the pressure is over 7.79 GPa, the radii of the Mo atom and Tm atom will become equivalent. In fact, during high-energy ball milling, a pressure of 7.79 GPa is not difficult to obtain and, in fact, the maximum pressure value *q*_0_ will be up to 8 GPa according to the following theoretical calculations.

Assuming that two impacting balls have the same elastic properties, and taking *γ* = 0.3, the formula is shown as follows [30]:

(21) $$\alpha =1.23\times \sqrt[{3}]{\frac{P^{2}}{E^{2}}\frac{R_{1}+R_{2}}{R_{1}R_{2}}}$$

(22) $$q_{0}=1.5\times \frac{p}{\pi a^{2}}=1.5\bar{q}=0.388\times \sqrt[{3}]{PE^{2}{\left(\frac{R_{1}+R_{2}}{R_{1}R_{2}}\right)}^{2}}$$

where *α* is the amount of elastic deformation between two colliding mill balls; *P* is the compression force under the colliding process; *E* is Young’s modulus; *R*_1_ and *R*_2_ are the radius of the colliding balls; *q*_0_ is the pressure at the center of the surface of contact, which is the maximum pressure of the contacting surface; and $\bar{q}$ is the average pressure on the contact surface of two colliding mill balls.

The velocity of the ball in the present work is taken as *υ* = 2 m/s. The weight of a mill ball is 0.51 g. The calculated impact energy is *E_k_* = 2 × 0.5 mυ^2^ = 0.0024 J. Therefore, the pressure *q* of two impacting balls can be obtained using the following integral:

(23) $$E_{k}=2\int_{0}^{\alpha /2}\;pdx$$

Using Equations (21) and (23), *E_k_* is: *E_k_* = 1.923 × 10^9^ α^5/2^. The compression force *P* can then be calculated and is *P* = 355.623 N. According to Equation (22), *q*_0_ = 8.369 GPa and $\bar{q}$ = 5.58 GPa.

Therefore, the value of *q*_0_ = 8.369 GPa is larger than that of q* = 7.79 Gpa. The Tm atoms are easily dissolved into the Mo crystal structure when the Tm atom radius is less than or equal to the Mo atom radius. Certainly, in the above discussion, the calculation is an ideal condition. In actuality, the collision of two balls is random and is not always a frontal collision because of random motion of the grinding balls in the can. The values of collisional pressure are in a Gaussian distribution. The pressure of a part of collision in the actual can be over the q* value, which will induce the radius of Tm atom to reach that of Mo atoms. Therefore, a long time is needed to make all Tm atoms dissolve into the Mo crystal structure. 

Simultaneously, the temperature at the colliding surface will instantaneously increase several hundred degrees Celsius, resulting from the impact of the balls, which promotes the diffusion and dissolution process of Tm atoms. Davis reported that the maximum temperature rise during ball milling was approximately 300 °C in a Fe (1.2 wt %)-C steel system [31]. Tonejc’s research showed that the local rise in temperature was at least 570 °C in a Ga-Sb alloy system [32]. Meanwhile, the large defect densities increase the free energies of atoms, which also promotes Tm atom dissolution. 

## 4. Conclusions

The microstructural characteristics of Mo-21%Tm_2_O_3_ (mass fraction, %) powder mixtures during ball milling were investigated by using transmission electron microscopy and X-ray diffraction. The thermodynamics and kinetics of phase evolution were also analyzed and discussed. The experimental results showed that the powder mixtures were first fined and nano-crystallized, and then transformed into supersaturated nanocrystalline solid solutions. The diffraction peaks of the Mo phase shifted to low angles during ball milling. The diffraction peaks of the Tm_2_O_3_ phase disappeared completely after 96 h of ball milling, which was attributed to ball milling-induced decomposition of the Tm_2_O_3_ component followed by solvation into the Mo crystal structure. The elements of Mo, Tm, and O were distributed uniformly in the ball-milled particles. After 96 h of ball milling, a supersaturated nanocrystalline solid solution of Mo (Tm, O) was formed. Based on the semi-experimental theory of Miedema, a thermodynamic model was developed to calculate the driving force of phase evolution. There was no chemical driving force to form a crystal solid solution of Tm atoms in the Mo crystal structure, or an amorphous phase, because the Gibbs free energy was higher than zero for both processes. For Mo-21%Tm_2_O_3_, it was mechanical work, not the negative heat of mixing, which provided the driving force to form supersaturated nanocrystalline Mo (Tm, O) solid solution. The current experimental results of the ball-milled powder mixtures can provide sufficient support for subsequent bulk preparation. The relationships between mechanical properties, thermophysical properties, physical efficiency, and irradiation properties of bulk materials and the characteristic microstructure of several kinds of ball-milled powders will be achieved in the future based on the current study results.

## Figures and Tables

**Table 1:** Thermodynamics parameters for calculation [27,28].

Element	P (KJ·V^−2^·cm^−1^)	Q (KJ·cm)	*n*^1/3^ (cm^−1^)	Ф (V)	K (10^10^ N·m^−2^)	μ (10^10^ N·m^−2^)	V (cm^3^·mol^−1^)	Tm (K)	Hf (KJ/mol)
Mo	12.35	132.54	1.77	4.65	26.12	12.56	9.4	2890.2	37.48
Tm	12.35	132.54	1.23	3.12	44.5	30.5	18.124	1818	16.8
